# Supplementary figures and images for: Kdm6a deficiency restricted to mouse hematopoietic cells causes an age- and sex-dependent myelodysplastic syndrome-like phenotype
Source: PLoS One. 2021 Nov 15;16(11):e0255706. doi: 10.1371/journal.pone.0255706 (PMC8592440; doi:10.1371/journal.pone.0255706)

Figure S1

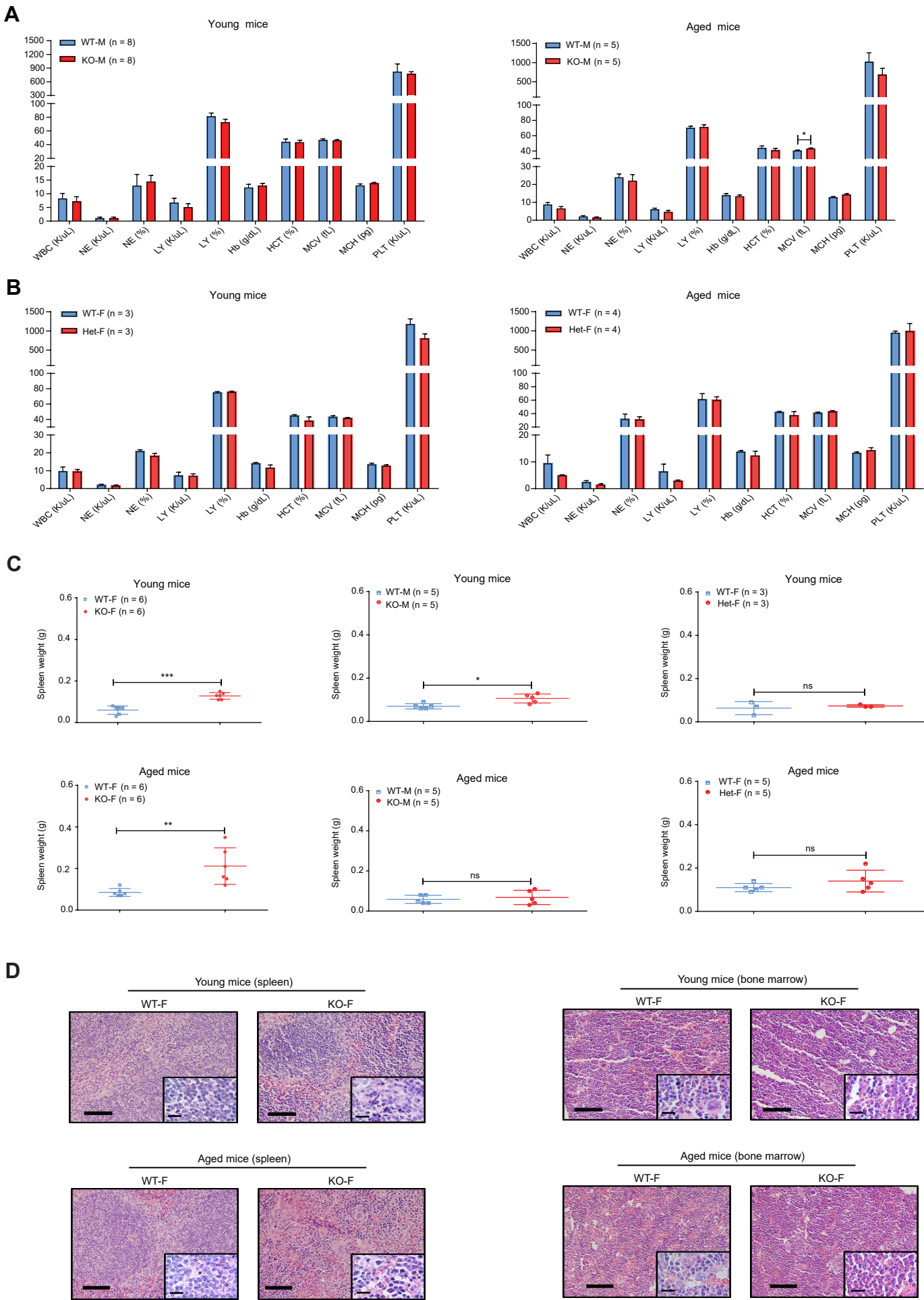

Supplement: S1 Fig — (A) CBCs for the young and aged KO-M mice versus WT-M mice showed no significant differences, except in aged mice which had an increase in MCV. (B) CBCs for the young and aged Het-F mice versus WT-F mice showed no significant differences. (C) Splenomegaly was present in young KO-F mice (0.13 g in KO-F mice versus 0.06 g in WT-F mice, p = 0.0001) and aged KO-F mice (0.212 g in KO-F mice versus 0.085 g in WT-F mice, p = 0.0062). We also observed mild splenomegaly in young KO-M mice (0.11 g in KO-M mice versus 0.07 g in WT-M mice, p = 0.0102), but no significant differences between other matched cohorts. (D) Histopathologic examination of BM and spleen by H&E staining. BM sections showed moderately to markedly increased numbers of myeloid cells and decreased numbers of erythroid cells from young to aged KO-F mice. The myeloid to erythroid ratio was typically elevated, up to 20:1 in some fields. Emperipolesis was often noted. Megakaryocytes were present in expected to increased numbers. Sections of spleen from KO-F mice showed extramedullary erythropoiesis and granulopoiesis that increased with age. Representative images (40x magnification) including small fields (100x magnification) are shown. Error bars represented mean ± s.d. *p<0.05, **p<0.01, ***p<0.001 by unpaired two-tailed Student’s t-test. (PDF) [file pone.0255706.s001.pdf]

Figure S2

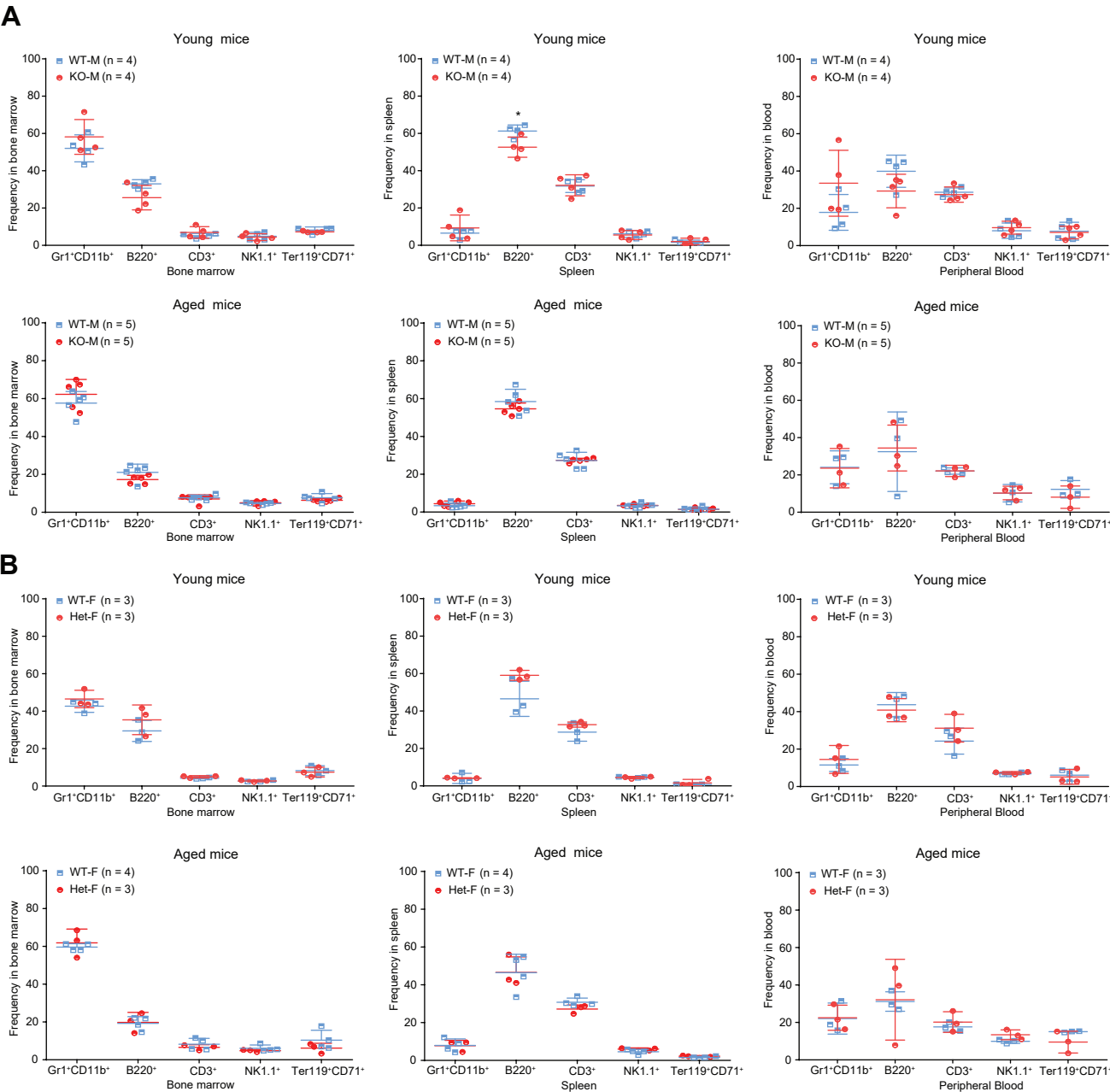

Supplement: S2 Fig — (A) Cell lineage frequencies for BM, spleen and PB from young and aged, KO-M and WT-M mice were determined by flow cytometry for myeloid cells, B-cells, T-cells, NK cells and erythroid precursors. There was a significant decrease in B220+ B cells in the spleen of young KO-M mice as compared to WT-M mice. (B) Cell lineage frequencies for BM, spleen and PB from young and aged, Het-F and WT-F mice were determined by flow cytometry for myeloid cells, B cells, T cells, NK cells and erythroid precursors. There were no significant differences observed in these compartments. Error bars represented mean ± s.d. *p<0.05 by unpaired two-tailed Student’s t-test. (PDF) [file pone.0255706.s002.pdf]

Figure S3

**A**

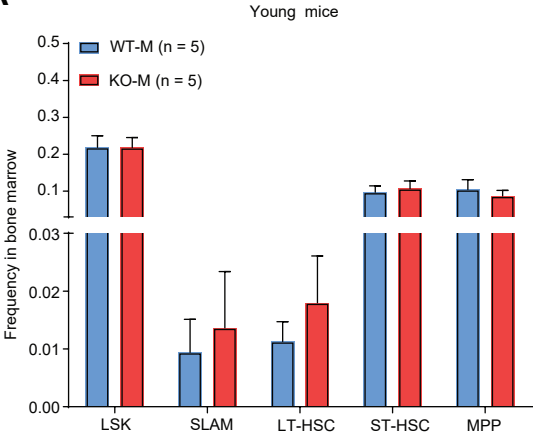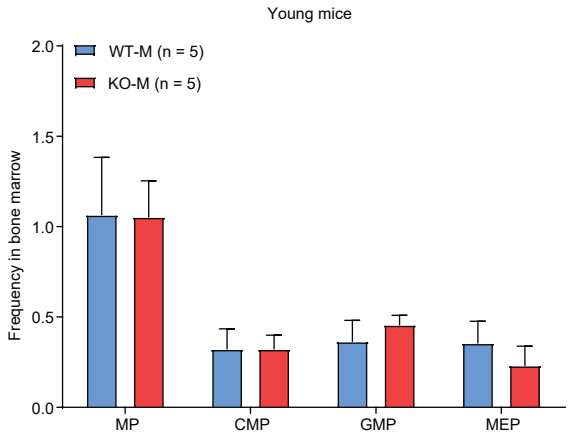

**B**

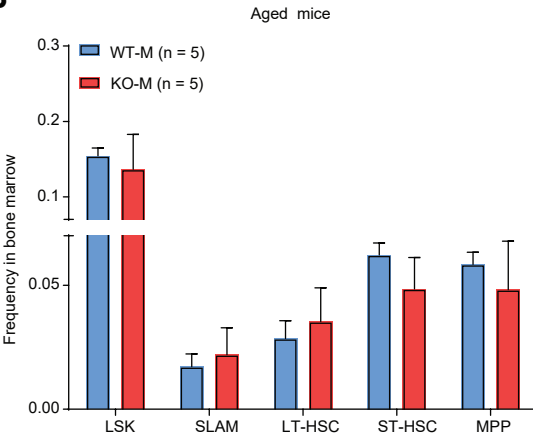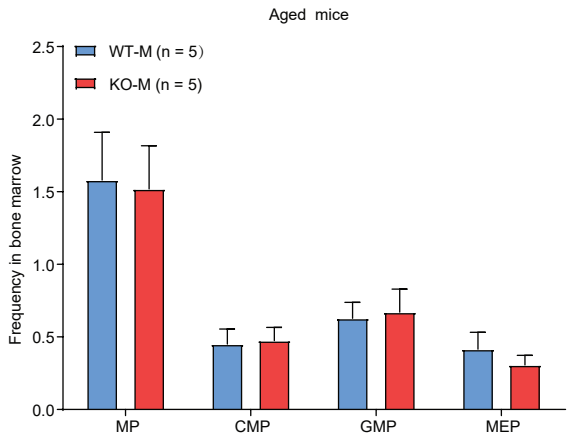

**C**

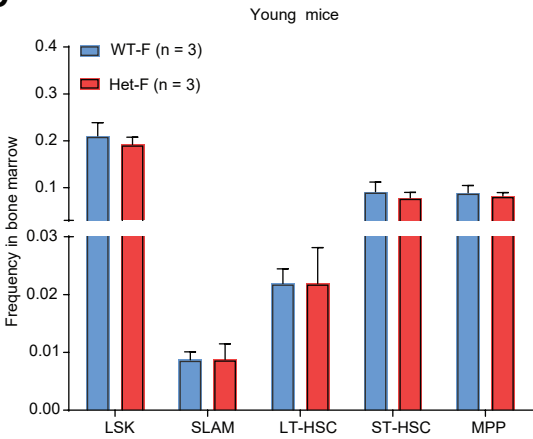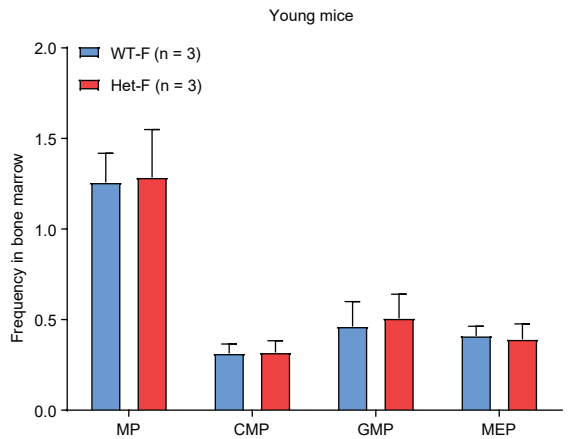

**D**

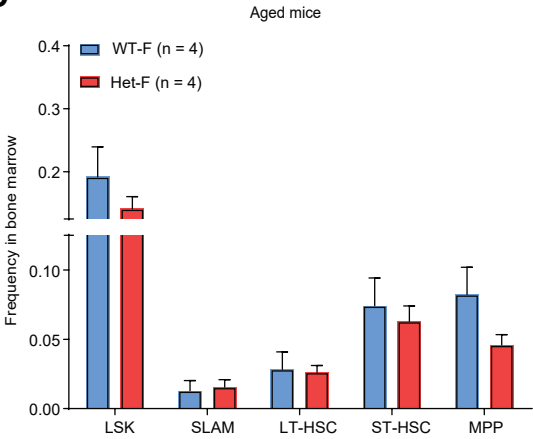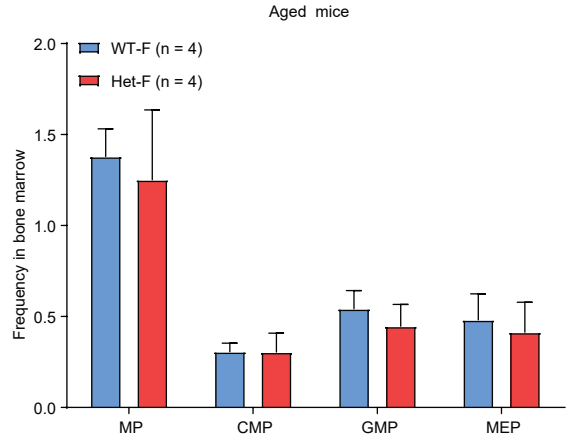

Supplement: S3 Fig — Flow cytometry analysis of HSPC populations in young and aged KO-M and Het-F mice (compared with appropriate littermate controls). Quantification of the indicated stem cell and progenitor compartments showed no differences for these genotypes at any time point. (PDF) [file pone.0255706.s003.pdf]

Figure S4

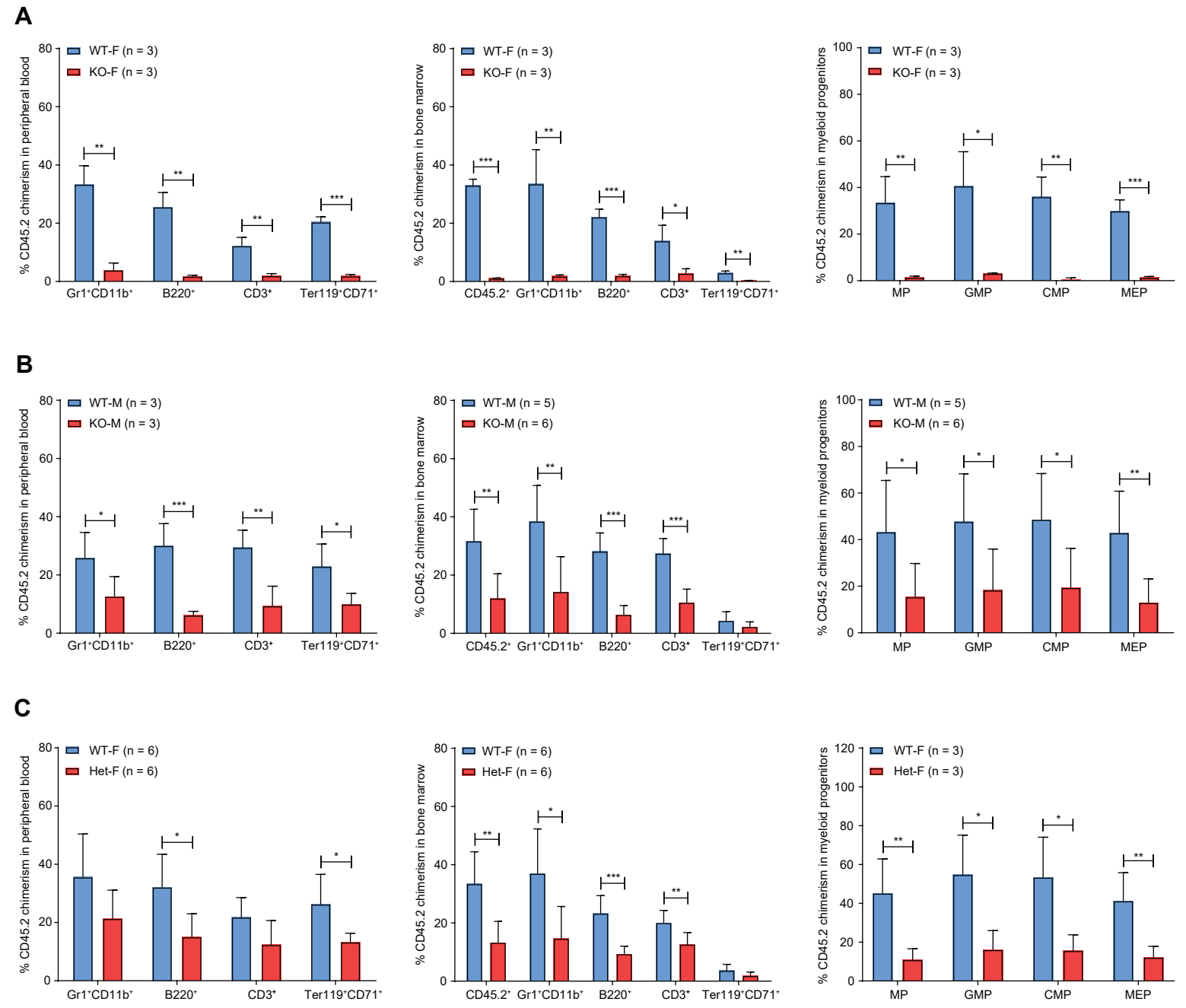

Supplement: S4 Fig — (A) Quantification of donor cell chimerism in mature cell lineage and myeloid progenitor cell populations each in the PB and BM analyzed by flow cytometry 36 weeks after transplantation showed a nearly complete loss of KO-F donor cells in these cell populations. (B) Quantification of donor cell chimerism in mature cell lineage and myeloid progenitor cell populations each in the PB and BM analyzed by flow cytometry 36 weeks after transplantation showed a significant decrease of KO-M donor cells in these populations. (C) Quantification of donor cell chimerism in mature cell lineage and myeloid progenitor cell populations each in the PB and BM analyzed by flow cytometry 36 weeks after transplantation showed a significant decrease of Het-F donor cells in these cell populations. Error bars represented mean ± s.d. *p<0.05, **p<0.01, ***p<0.001 by unpaired two-tailed Student’s t-test. (PDF) [file pone.0255706.s004.pdf]

Figure S5

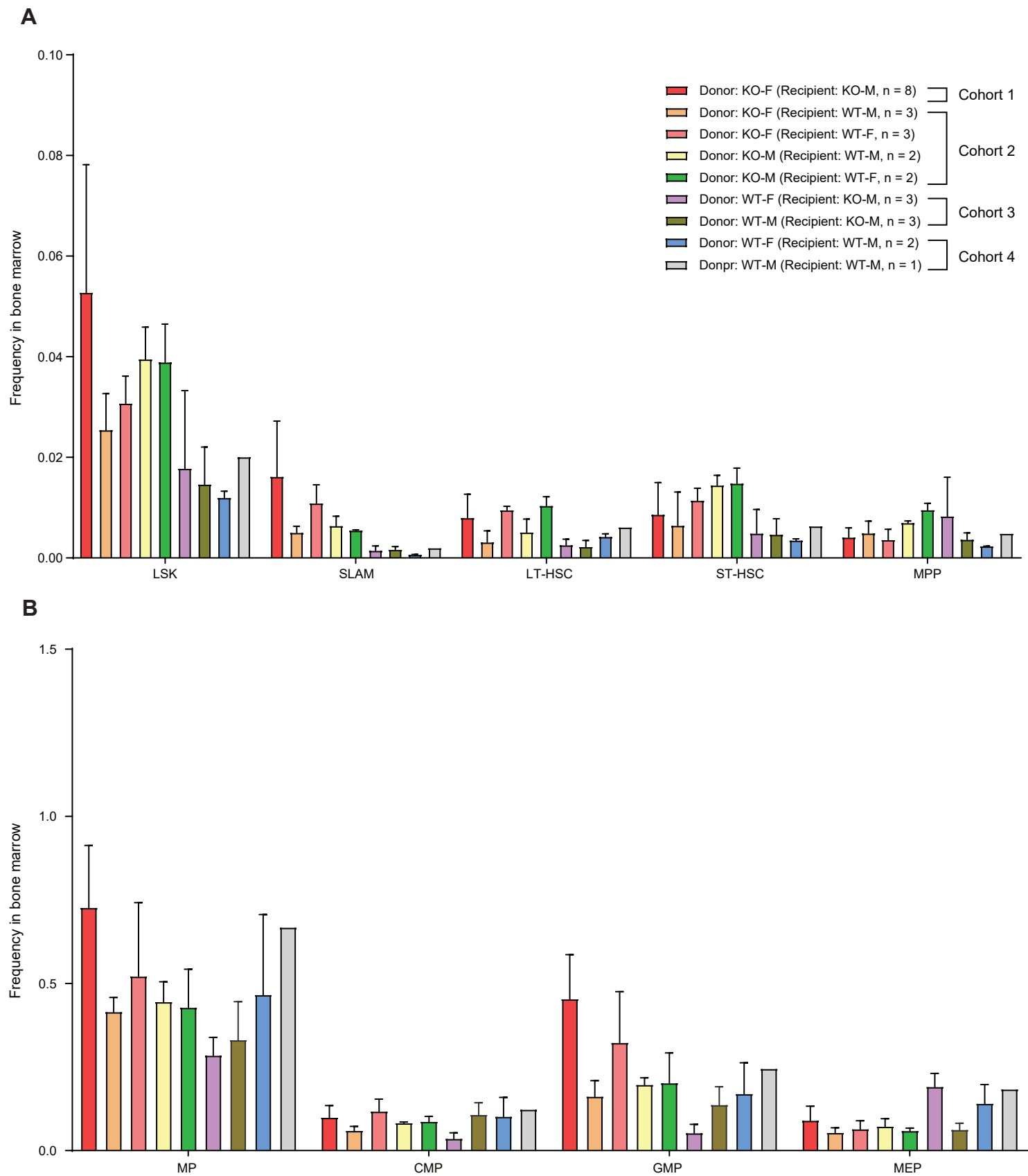

Supplement: S5 Fig — (A) Quantification of the indicated stem cell compartments for cohorts 1–4, separated by sex. (B) Quantification of the indicated progenitor compartments for cohorts 1–4, separated by sex. (PDF) [file pone.0255706.s005.pdf]

Figure S6

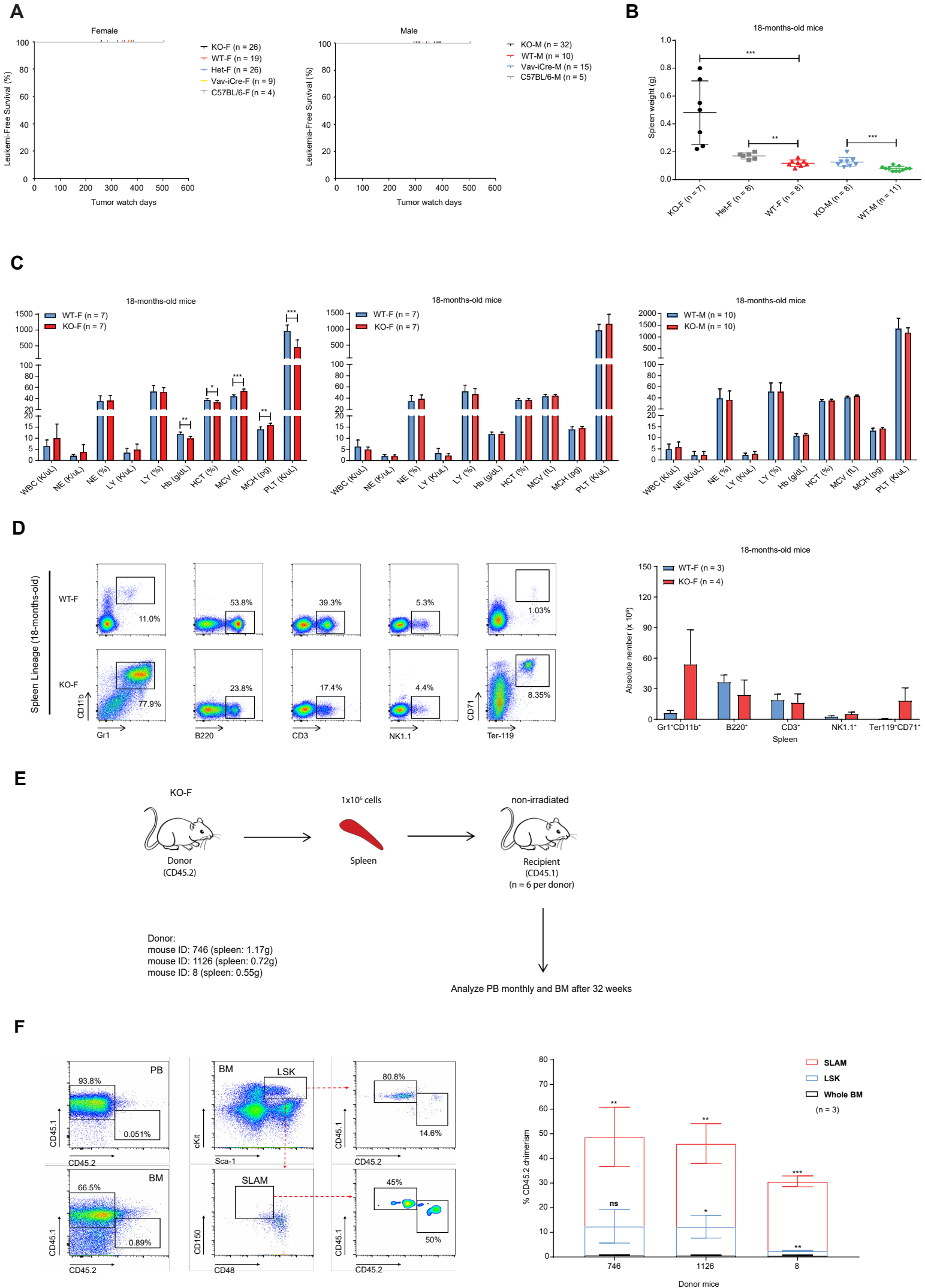

Supplement: S6 Fig — (A) The normal survival curve demonstrated that the inactivation of Kdm6a could not induce mice to develop overt leukemia or die of lethal abnormalities. (B) Spleen weight for all genotypes of Kdm6a conditional KO mice and appropriate littermate controls. Several KO-F mice had marked splenomegaly (> 0.5 g). (C) CBCs showed that KO-F mice had anemia and thrombocytopenia, but no difference was seen in Het-F or KO-M mice. (D) Flow cytometry analysis of mature cell lineage in the spleen from KO-F and WT-F mice. Representative flow cytometry plots showed the deletion of Kdm6a caused a striking myeloid skewing with an increased number of Gr1+CD11b+ myeloid cells and an increase in Ter119+CD71+ erythroid precursors with age. (E) Experimental scheme of the KO-F spleen cells transplantation. Spleen cells were harvested from 3 18 months old KO-F donor mice and transplanted into non-irradiated recipient mice. PB was examined for donor cell chimerism on a monthly basis after transplantation. (F) PB donor cell chimerism indicated the donor-derived spleen cells could not engraft in recipient mice. After long-term observation, mice from each donor cohort were sacrificed and BM cells were harvested for flow cytometry analysis. Representative flow cytometric plots and quantification of donor cell chimerism in whole BM and the HSC compartment (LSK). KO-F donor HSC cells engraft and exhibited a great contribution to the SLAM compartment (14.6% in LSK cells and 50% in SLAM cells). Error bars represented mean ± s.d. *p<0.05, **p<0.01, ***p<0.001 by unpaired two-tailed Student’s t-test. (PDF) [file pone.0255706.s006.pdf]

Figure S7

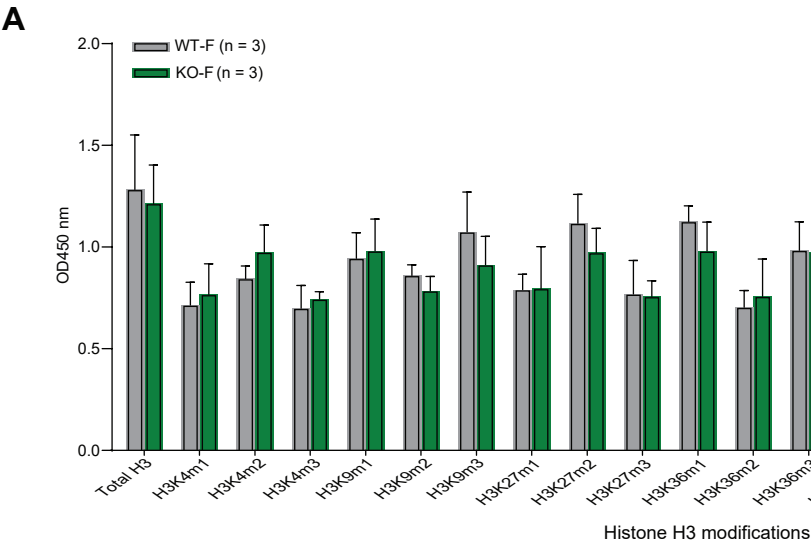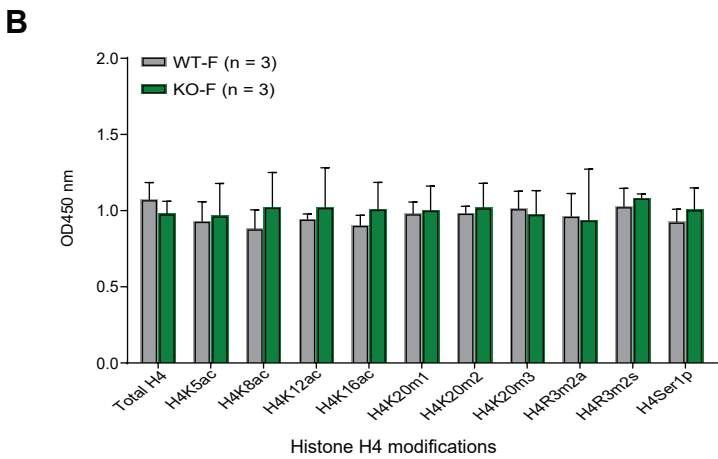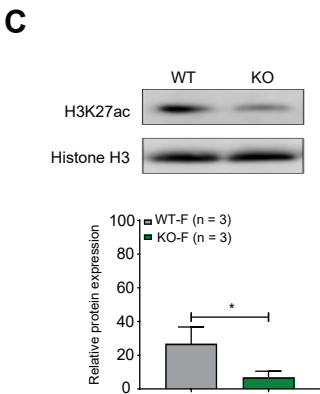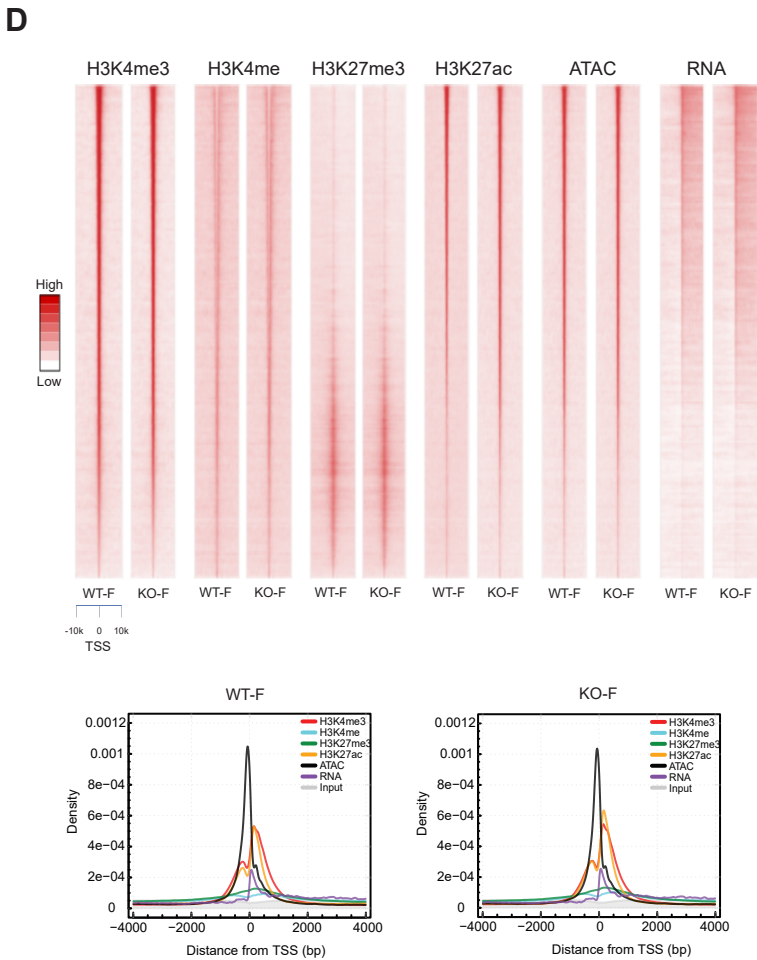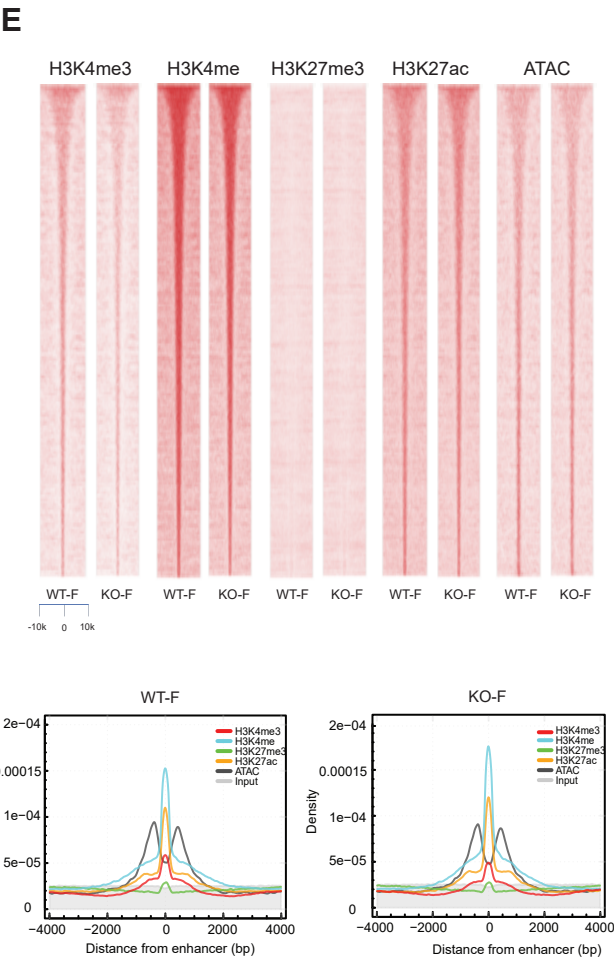

Supplement: S7 Fig — (A) The detection and quantification of 21 different histone H3 modifications using total core histone proteins extracted from WT-F and KO-F mice whole BM cells. (B) The detection and quantification of 10 different histone H4 modifications using total core histone proteins extracted from WT-F and KO-F mice whole BM cells. (C) Western blot analysis of core histone protein lysates extracted from whole BM cells showed a decrease of H3K27 acetylation in the young KO-F mice compared with WT-F mice. (D) Peak density heat maps for each ChIP-seq dataset, ATAC-seq and bulk RNA-seq around the transcription start sites (TSSs) of the complete set of 18,624 potentially transcriptionally-engaged promoters of annotated genes defined by our own H3K4me3 ChIP-seq dataset. Total peaks detected for H3K27ac were 33,464 peaks (139,420,356 reads) for WT-F samples and 28,290 peaks (146,408,232 total reads) for KO-F samples. Tag densities within a window of +/− 10 kb on either side of TSS coordinates were collected from each dataset. We observed the expected inverse relationship between H3K4me3 and H3K27me3 peak density that correlates with transcriptional activity of annotated genes (defined by bulk RNA-seq data) (top panel). Peak density profiles around TSS (+/− 4kb) for each ChIP-seq dataset with ATAC-seq and bulk RNA-seq (lower panel). (E) Peak density heat maps for each ChIP-seq dataset and ATAC-seq around enhancer-like regions of the complete set of 14,892 enhancer-like regions by presence of H3K4me peaks and ATAC-seq. Tag densities within a window of +/− 10 kb on either side of the enhancer coordinates were collected from each dataset (top panel). The peak density profiles around enhancers (+/− 4kb) for each ChIP-seq dataset with ATAC-seq (lower panel). Error bars represented mean ± s.d. *p<0.05 by unpaired two-tailed Student’s t-test. (PDF) [file pone.0255706.s007.pdf]

Figure S8

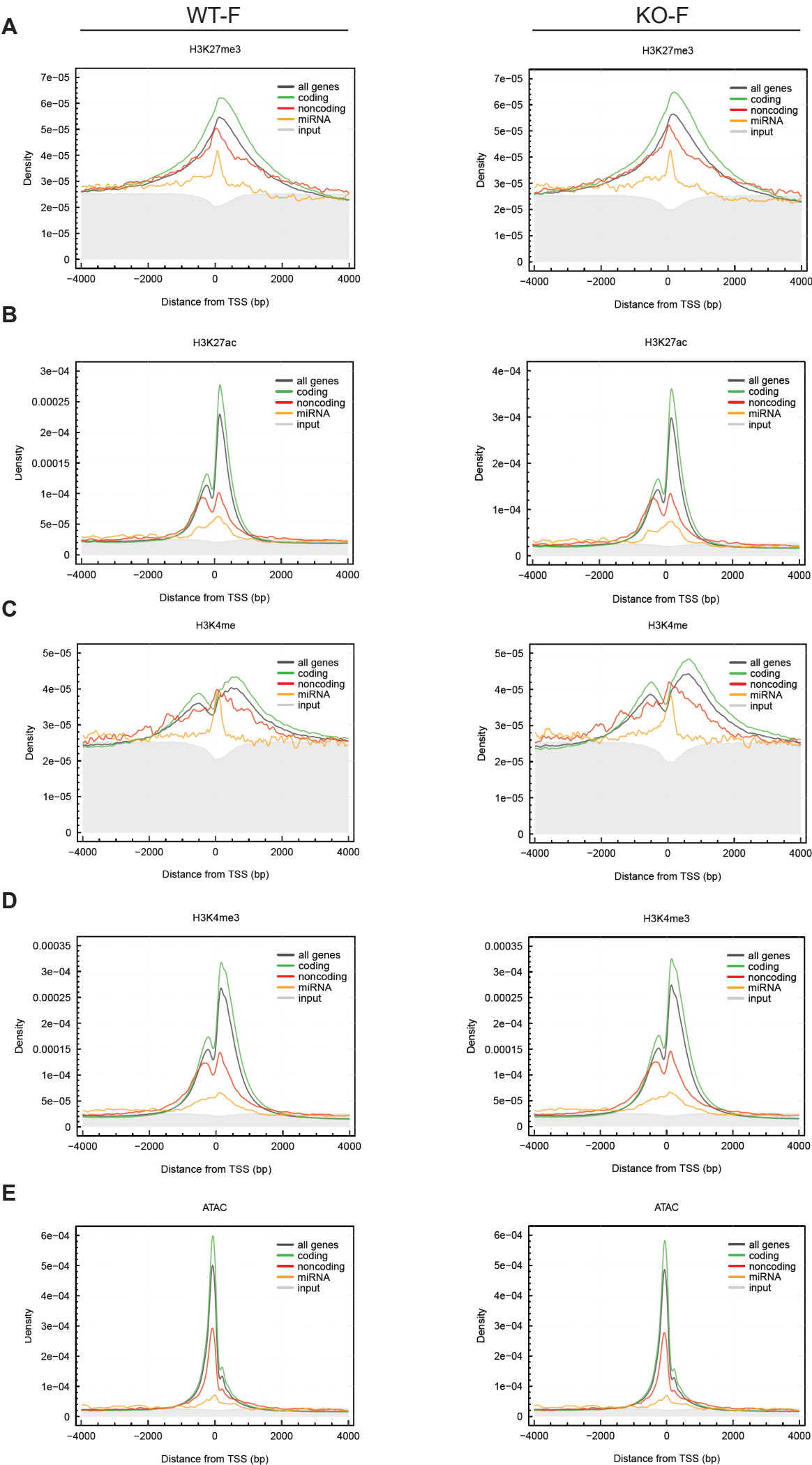

Supplement: S8 Fig — (A) H3K27me3 ChIP-seq (n = 3 per genotype). (B) H3K27ac ChIP-seq (n = 2 per genotype). (C) H3K4me ChIP-seq (n = 2 per genotype). (D) H3K4me3 ChIP-seq (n = 3 per genotype). (E) ATAC-seq (n = 2 per genotype). ChIP-seq and ATAC-seq using bulk BM cells derived from young, KO-F mice versus young, WT-F mice as controls. (PDF) [file pone.0255706.s008.pdf]
