# Supplementary material for: Kdm6a deficiency restricted to mouse hematopoietic cells causes an age- and sex-dependent myelodysplastic syndrome-like phenotype
Source: PLoS One. 2021 Nov 15;16(11):e0255706. doi: 10.1371/journal.pone.0255706 (PMC8592440; doi:10.1371/journal.pone.0255706)
Supplement: S1 Methods — (DOCX) [file pone.0255706.s013.docx]

**Supplementary Methods**

**Mouse generation and characterization**

We characterized normal hematopoiesis from young (<12 weeks) and aged (50- to 55- week-old) *Kdm6a* conditional KO mice. For dissection, mice were euthanized by CO2 asphyxiation. Peripheral blood (PB), bone marrow (BM) and spleen were collected. For eye bleeding, PB was taken from anesthetized mice. A complete blood count (CBC) of PB was measured by an automated counting instrument (Hemavet 950; Drew Scientific Group). BM cells were harvested by flushing femurs and tibias. Isolated whole spleen was homogenized using 70-μm Nylon Cell Strainer (Corning™ 431751) to obtain a single cell suspension. We also kept track of body and spleen weights. We established tumor watch of all animals noted above. The mice were taken down after long-term observation (18 months). Diagnosis of leukemic-like disease was conducted by CBC, flow cytometry and pathology. The Kaplan-Meier survival curve was analyzed by Prism 7 (GraphPad Software).

**Haematoxylin Eosin (H&E) staining**

Fresh femurs and spleen were collected from young and aged WT-F and KO-F mice. Rinsed with PBC buffer, then fixed with 10% formalin for 24 hours at room temperature. H&E staining was processed by DCM Research Animal Diagnostic Laboratory, Washington University in St.louis School of Medicine.

**Giemsa-Wright staining**

For cytospin preparation spin 200 ul of FACS (1 X DPBS, 5% FBS, 0.5 M EDTA) buffer onto a slide (1000 rpm, 2 minutes). Next, spin and resuspend 150,000 fresh BM cells harvested from aged –WT-F and KO-F mice in 200 ul of FACS buffer. Spin onto the slide at a slow acceleration (350 rpm, 7 minutes). For staining, mix a 9:1 ratio of SAS-100 buffer (pH 6.8) to Giemsa stain and let stand for 10 minutes. Place 2 ml wright stain upon the cells, in a sufficient quantity to cover the entire surface for 5 minutes. Dump off liquid, and quickly add 4 ml Geimsa + buffer mix for 10 minutes. Rinse with DI water in sink for 20 seconds and allowed to dry in the air.

**Western blotting**

To make a protein lysate 250,000 whole BM cells were rinsed with PBS and lysed in urea lysis buffer (7 M urea, 2 M thiourea, 30 mM Tris, pH 8.5) supplemented with protease inhibitor cocktail (Abcam, ab65621) and then snap frozen in liquid nitrogen. The protein lysate was mixed with 6x loading buffer (0.01 M Tris-HCl, pH 6.8, 8% glycerol, 0.1 mg/mL bromophenol blue, 2% SDS, 1% β-mercaptoethanol) and was boiled for 8 minutes at 100 °C.

Protein lysate was separated on a precast gel (BIO-RAD) and transferred onto PVDF blotting membrane (Amersham, GE Healthcare). The membrane was probed with anti-UTX (1:500; BETHYL; catalog A302-374A); anti-Histone H3K27ac (1:1000; ACTIVE MOTIF; catalog 39133); anti-Histone H3, C-terminal (1:10,000; ACTIVE MOTIF; catalog61277); or anti-β-Actin (1:10,000; Cell Signaling Technology; catalog 3700s). Immune complexes were revealed by peroxidase linked anti-mouse IgG (1:10,000; GE Healthcare; catalog NA931V) or peroxidase linked anti-rabbit IgG (1:10,000; GE Healthcare; catalog NA934V). The western blots were visualized by chemiluminescence using ECL Prime Western Blotting Detection Reagent (Amersham, GE Healthcare) and a ThermoFisher myECL Imager (Thermo Fisher Scientific).

**Transplantation assays**

Competitive BM transplantation was performed using 0.5 x 10^6^ whole BM cells from indicated 6- to 8- week-old donor mice (CD45.2) mixed with 0.5 x 10^6^ competitor whole BM cells from 6- to 8- week-old WT mice (CD45.1 X CD45.2). Total BM cells were injected intravenously into 6-week-old lethally-irradiated recipient mice CD45.1 (NCI B6-Ly5.1/Cr, Charles River) that received 1,100 cGy body irradiation (Mark 1 Cesum^137^ irradiator, JL Shepherd) 24 hours prior to transplantation. For secondary transplantation, 1 x 10^6^ whole BM cells harvested from primary recipient mice were injected into secondary lethally-irradiated recipient mice (CD45.1).

For Kdm6a conditional KO x Cre-ERT2 transplantation, 1 x 10^6^ whole BM cells from indicated 6- to 8-week-old donor mice (CD45.2) were injected intravenously into 6- to 8-week-old lethally irradiated indicated recipient mice (CD45.2) that received 1,100 cGy body irradiation 24 hours prior to transplantation. Cre expression was induced by tamoxifen (CAYMAN CHEMICAL) 6 weeks post-transplant via oral gavage for 9 doses total (3 mg/day/mouse, 3 days/week).

For adoptive transfer experiment, 1 x 10^6^ spleen cells harvested from 3 different 18-months-old donors (CD45.2) which had spleens bigger than 0.5 g were injected intravenously into 6-week-old non-irradiated recipient mice (CD45.1).

**Cell cycle and apoptosis assays**

For cell cycle analysis mice whole BM cells were harvested from indicated mice and suspended in warm transplant medium and incubated with 10 μM EdU for 2 hours, followed by a 30 minute incubation with Lin^-^Sca-1^+^c-Kit^+^ (LSK) cell population cell surface makers on ice. After fixation and permeabilization, the cells were stained with FxCycle Violet DNA dye (Life Technologies) for 30 minutes on ice. Flow cytometry was performed by Gallios flow cytometer (Beckman Coulter Life Sciences).

# For apoptosis analysis whole BM cells were harvested and suspended in FACS buffer, incubated with LSK cell population cell surface makers for 30 minutes on ice, and washed twice and resuspended in 1X Binding Buffer. 1 x 10^6^ cells per 100 μl were then stained with Annexin V (FITC) and PI using FITC Annexin V Apoptosis Detection Kit (BD Biosciences) for 15 minutes at room temperature in the dark. 400 µl of 1 X Binding Buffer was added to each tube and analyzed by Gallios flow cytometer (Beckman Coulter Life Sciences).

**qPCR**

The Applied Biosystems Kdm6a floxed primers are: forward primer (TGGCACCTTAGATTCTTGAACTGAT); reverse primer (GCGCCGGAACCGAAGT). Probe is labeled with FAM dye-MGB and the sequence is AGCTCAGACCATAACTTCGTATA. The Applied Biosystems Mouse ACTB (VIC-MGB Probe, Primer Limited) was used as an endogenous control.

**Histone H3 or Histone H4 modifications**

The total core histone proteins were extracted from whole BM cells from indicated mice by using the EpiQuik Total Histone Extraction Kit (EPIGENTEK). 21 different histone H3 modifications and 10 different histone H4 modifications were measured simultaneously by the EpiQuik™ Histone H3 or H4 Modification Multiplex Assay Kit (EPIGENTEK). The ratio of modified histone was proportional to intensity of absorbance measured by Epoch Microplate Spectrophotometer (BioTek).

**Bulk RNA-Sequencing, processing, and analysis**

Whole BM cells from <12-week-old KO-F and WT-F mice were used for bulk RNA-sequencing (RNA-seq). RNA was isolated using Zymo MicroRNA Kit (ZYMO RESEARCH). Total RNA library was prepared using the TruSeq Stranded Kit (I[llumina](https://www.illumina.com/products/by-type/sequencing-kits/library-prep-kits/stranded-mrna-prep.html)), sequenced by 125-bp read length on the Illumina HiSeq platform and aligned to the mouse reference sequence (NCBI37/mm9 build)^1^. Tophat version 2.0.8 ^2^ was used for read mapping. We used these resulting BAMs for manual review of RNA-seq data by using the Integrated Genomics Viewer (IGV). Kalisto version 0.44.0 was used for quantifying the transcript-level abundance from raw sequencing data with the gene annotation from Ensembl Release 67 of Mus musculus (NCBIM37). We used edgeR (Empirical Analysis of Digital Gene Expression Data in R) version 3.22.2 in the Bioconductor package for differential expression analysis at the gene level. Transcript-level read counts were calculated by Kalisto quantification algorithm and summarized for gene-level expression analysis using the Bioconductor package tximport version 1.8.0. R Package ‘pheatmap’ version 1.0.12 was used for visualizing gene expression data.

**Histone Methylation Chromatin Immunoprecipitation-Sequencing (ChIP-seq), Transposase-Mediated Chromatin Accessibility Profiling (ATAC-seq), and data analysis**

Fresh whole BM cells were harvested from <12-week-old WT-F and KO-F mice. ChIP-seq was performed with the MAGnify ChIP kit (Thermo Fisher) as described by Spencer DH *et al*.^3^ using a total of 1-1.5 million cells per sample, which were distributed across [immunoprecipitation](https://www.sciencedirect.com/topics/neuroscience/immunoprecipitation) reactions for [histone](https://www.sciencedirect.com/topics/biochemistry-genetics-and-molecular-biology/histone) H3K4me (Abcam, ab8895), H3K4me3 (Abcam, ab8580), H3K27me3 (Active Motif, 39155) and H3K27ac (Active Motif, 39133). Library molecules were prepared using the NEBNext Library Prep Kit (BioLabs) for Illumina, quantified and QC’ed via Qubit and Agilent Bioanalyzer 2100 analysis. ATAC-seq was performed with the Nextera Library Prep (Illumina) as described by Buenrostro JD *et al* ^1^. using 75,000-150,000 viable cells per sample. Libraries were size-fractionated (100-800 bp) and 15 Gb/sample for Illumina, quantified and QC’ed via Qubit and Agilent Bioanalyzer 2100 analysis. Sequencing was performing in the paired-end mode on the Illumina HiSeq platform. Raw sequencing data were aligned to the mouse reference sequence (NCBI37/mm9 build) by using BWA version 0.5.9 ^4^ with the parameters (-t 4 -q 5::), then merged and deduplicated using Picard version 1.46 (<https://broadinstitute.github.io/picard/>).

For data analysis, we first assessed and ensured ChIP-seq and ATAC-seq reproducibility by biological replicate experiments from independent mice pools. We performed replicate consistency/correlation analysis and manual review of ChIP-seq and ATAC-seq data using IGV ^5^ for data inspection. Next, each biological replicate data and their combined data per histone modification were analyzed for the following integrative analysis, given replicate criteria. The Model-based Analysis of ChIP-Seq (MACS) version 1.4.2 ^6^ was used to identify genomic regions of histone modification enrichment by ChIP-seq and chromatin accessibility by ATAC-seq. For ChIP-seq data processing the middle position from properly paired reads on the same chromosome was calculated and compiled as tag indices ^7^. If any reads were marked as PCR or optical duplicates in the SAM FLAG field, they were filtered out. We also filtered out any tag indices if they were in the blacklisted genomic regions of the mouse genome curated for functional genomics analysis ^8^. Resulting tag indices of each replicate of histone ChIP-seq were fed to the peak calling algorithm (MACS version 1.4.2) with the tag indices from ChIP input data as the default parameters. We upgraded MACS software for the purpose of applying a new input format of tag indices and optimized the algorithms to define the peak boundary accordingly. Next, we counted the number of tag indices given the boundary of each peak call, as the count number of sequencing templates or ChIP'ed DNA fragments for sequencing. This quantity was the basis of measuring the occupancy or enrichment level of chromatin with the given histone modification. The peak summit position that MACS reported from each peak call was used as the reference genomic coordinate of those peaks in the downstream analysis, defined as the position of the most tag indices (i.e. sequencing templates) in the peak call. We normalized the count numbers between replicates using the total count number for data visualization and inspection purpose.

For ATAC-seq data process, we shifted the start coordinate of each sequencing read in order to represent the center of the transposon binding event in the assay, which was described previously ^1^. From the properly paired reads on the same chromosome, any reads aligned to the forward strand (+) were offset by +4 bp, and all reads aligned to the reverse strand (-) by −5 bp, and those positions were compiled as tag indices for ATAC-seq. The same data process (as used for histone ChIP-seq data analysis) was applied for ATAC-seq peak calling using MACS version 1.4.2, but without control data files. Since those tag indices are the locations where the Tn5 transposase inserts sequencing adapters into accessible regions of chromatin, the tag index counts were used as accessibility measurement of open chromatin.

**Integrative Epigenetic Analysis for Identification of Active Regulatory Regions**

We determined two active regulatory genomic elements by integrating H3K4me, H3K4me3, H3K27me3, H3K27ac ChIP-seq, and ATAC-seq with known gene annotation: active enhancer regions and active transcription start sites (or TSS’s) in transcriptionally engaged chromatin. We performed integrated genomic analysis by using known epigenetic features of enhancers ^9^ for the identification of active promoter-distal enhancers of the mouse models used in this study. We defined enhancer candidates in a primed state by using our H3K4me ChIP-seq data ^10^. We called peaks by combining all H3K4me replicates to define the boundary of “consensus” H3K4me enrichment regions and their consensus peak summits. We used only the consensus H3K4me peak calls that passed the biological replicate criteria of having peak calls from at least 1 biological replicate (of total n = 2) present in either WT-F or KO-F mice within 1,000-bp peak summit-to-summit distance. The peak summit from the consensus peak calls (defined by MACS peak calling algorithm as of the most sequencing template count) were used as the reference genomic coordinate for that consensus location. We associated every consensus location with the count number of sequencing templates that were calculated from the combined H3K4me data, given the consensus peak boundary called by MACS. We interpreted this count number as the occupancy or enrichment level of H3K4me. We used a percentile rank as a cutoff threshold, by sorting consensus locations by that count number. We started from 55,503 consensus H3K4me enriched regions to define enhancer regulatory elements. We called consensus locations in the same manner with 150-bp distance threshold for ATAC-seq, 500 bp for H3K4me3 and H3K27ac, and 1,000 bp for H3K27me3. From the 50,503 enhancer candidates, we required less than 2,500-bp distance from consensus ATAC-seq peak calls and we filtered out any candidate if they were promoter-proximal (i.e. < 2,500 bp) using known TSS’s in the mouse gene annotation (Ensembl Release 67 of Mus musculus), except for miRNA genes. Subsequently, we filtered out any candidates that were in less than 2,500 bp distant from consensus H3K4me3 peak calls, whose occupancy is in the top 60% percentile among all consensus H3K4me location. This filtration step allowed us to further remove any candidates in the promoter regions of unannotated genes. As a result, 14,892 promoter-distal enhancer candidates were kept, whose H3K4me occupancy level was in the top 60% percentile. Among these enhancer-like candidates, H3K27ac peak proximity cutoff was placed as less than 2,000-bp distance for active enhancer candidates. We used consensus H3K27ac-enriched locations whose occupancy level was in the top 60% percentile. Then, we filtered out 79 candidates based on H3K27me3 proximity in less than 2,500-bp distance, and we finally listed 5,559 enhancer candidates as active enhancers in the downstream data analysis^11,12^. We intersected them with the published lists of known or putative enhancers and VISTA Enhancer Browser ^13^ and manually reviewed known enhancer regions via visual inspection in an integrated genome browser with the ChIP-seq and ATAC-seq data.

We employed an integrative approach with our histone ChIP-seq of H3K4me3 and the mouse gene annotation (Ensembl Release 67 of Mus musculus) for identification of TSS’s of the mouse models in transcriptionally engaged chromatin ^14^. First, we defined the boundary of the chromatin regions associated with H3K4me3 at the 5’ end of genes as the 2,000-bp region that is upstream -850 and downstream 1,150 bp relative to the TSS. We determined this boundary by calculating a composite distribution of H3K4me3 sequencing templates aligned by all annotated TSS’s ^15^. This H3K4me3 region was used to identify the genes with transcriptionally engaged promoters. Most mouse genes utilize many alternative TSS’s, so we first grouped annotated TSS’s per gene in the iterative manner, if their pairwise distance is less-than 250 bp away. Transcription unit grouping was used because of the broad resolution of histone ChIP-seq that could not resolve TSS’s that were close. From the H3K4me3 enrichment region, we calculated the count number of sequencing templates as CPM per biological replicate of H3K4me3 ChIP-seq data. We chose a TSS per each transcription unit group with the most CPM after summing up all biological replicates, then requiring at least any 1 consensus peak calls present (<2,500 bp) of either WT-F or KO-F mice. After removing noncoding genes whose biotype was snRNA, snoRNA, rRNA, Mt_tRNA, Mt_rRNA, and misc_RNA, we used 18,624 TSS’s in further downstream analysis.

**Flow cytometry sorting and single-cell RNA sequencing**

As previous described ^16,17^, fresh whole BM cells were harvested from <12-week-old WT-F or KO-F mice and resuspended in PBS with 2% FBS and 250 μM EDTA pH 8.0 at a concentration of 20 x 10^6^ cell/ml. Cells were then filtered through a 70-μm Nylon Cell Strainer into a 5-ml flow tube and stained with 1 μl Sytox red (Invitrogen) per 1 ml of cells for 15 minutes at 4 °C. Live cell sorting (sytox red negative) was performed on a modified Sony Synergy SY3200 (Sony Biochnology, San Jose, CA) updated to 24 parameters. Winlist version 8 software was used for data acquisition and analysis (Verity Softwar house, Topsham, ME). Cells were processed using the 10X Genomics Chromium Controller and the Chromium Single Cell 5′ Library & Gel Bead Kit (v2.0) (10X GENOMICS) following the standard manufacturer’s protocols (<https://tinyurl.com/y96l7lns>). Two WT-F vs two KO-F were then sequenced on the Illumina NovaSeq (2 x 150 paired end reads). Four HDF5 files were uploaded to the Partek server and used for downstream analysis. Quality analysis was performed using the Single-cell QA/QC task filter based on total reads (Min: 499, Max: 27826), expressed genes (Min: 100, Max: 4000), and mitochondrial counts (less than 10%). The normalization scheme used was counts per million (CPM), add 1, and Log2. The normalized data was filtered to remove features not expressed in any cells. Utilizing the principal components analysis (PCA) and the generated scree plot we chose the top 20 principal components for graph-based clustering and tSNE plot generation. The clustering algorithm used was Louvain at a 0.7 resolution. WT-F and KO-F classifications were used for analysis of variance (ANOVA) and the features with an FDR step up of less or equal to 0.01 or fold change of < -2 or > 2 were utilized for downstream analysis.

**Statistical Data Analysis**

For statistical differential analysis with histone modification in active regulatory regions between WT-F and KO-F mice, we used edgeR (Empirical Analysis of Digital Gene Expression Data in R) version 3.22.2 ^18^ in the Bioconductor package. For active enhancer regions, we calculated the counts of sequencing templates from ChIP-seq data of H3K4me, H3K4me3, H3K27me3, H3K27ac, and ATAC-seq, per each biological replicate of WT-F and KO-F mice. For this counting, we applied a fixed window of 3,000 bp in length, centered at the peak summit of consensus locations of H3K4me that was determined as an active enhancer region. We used edgeR to statistically analyze these count numbers per each ChIP-seq and ATAC-seq, and we listed active enhancer regions where that histone modification or chromatin accessibility (ATAC-seq) were differential between WT-F and KO-F mice at the FDR less than 5%.

**Supplemental References**

1. Buenrostro JD, Giresi PG, Zaba LC, Chang HY, Greenleaf WJ. Transposition of native chromatin for fast and sensitive epigenomic profiling of open chromatin, DNA-binding proteins and nucleosome position. *Nat Methods*. 2013;10(12):1213-1218.

2. Trapnell C, Pachter L, Salzberg SL. TopHat: discovering splice junctions with RNA-Seq. *Bioinformatics*. 2009;25(9):1105-1111.

3. Spencer DH, Russler-Germain DA, Ketkar S, et al. CpG Island Hypermethylation Mediated by DNMT3A Is a Consequence of AML Progression. *Cell*. 2017;168(5):801-816 e813.

4. Li H, Durbin R. Fast and accurate short read alignment with Burrows-Wheeler transform. *Bioinformatics*. 2009;25(14):1754-1760.

5. Robinson JT, Thorvaldsdottir H, Winckler W, et al. Integrative genomics viewer. *Nat Biotechnol*. 2011;29(1):24-26.

6. Zhang Y, Liu T, Meyer CA, et al. Model-based analysis of ChIP-Seq (MACS). *Genome Biol*. 2008;9(9):R137.

7. Kharchenko PV, Tolstorukov MY, Park PJ. Design and analysis of ChIP-seq experiments for DNA-binding proteins. *Nat Biotechnol*. 2008;26(12):1351-1359.

8. Consortium EP. An integrated encyclopedia of DNA elements in the human genome. *Nature*. 2012;489(7414):57-74.

9. Calo E, Wysocka J. Modification of enhancer chromatin: what, how, and why? *Mol Cell*. 2013;49(5):825-837.

10. Heintzman ND, Stuart RK, Hon G, et al. Distinct and predictive chromatin signatures of transcriptional promoters and enhancers in the human genome. *Nat Genet*. 2007;39(3):311-318.

11. Creyghton MP, Cheng AW, Welstead GG, et al. Histone H3K27ac separates active from poised enhancers and predicts developmental state. *Proc Natl Acad Sci U S A*. 2010;107(50):21931-21936.

12. Natoli G, Andrau JC. Noncoding transcription at enhancers: general principles and functional models. *Annu Rev Genet*. 2012;46:1-19.

13. Visel A, Minovitsky S, Dubchak I, Pennacchio LA. VISTA Enhancer Browser--a database of tissue-specific human enhancers. *Nucleic Acids Res*. 2007;35(Database issue):D88-92.

14. Bernstein BE, Kamal M, Lindblad-Toh K, et al. Genomic maps and comparative analysis of histone modifications in human and mouse. *Cell*. 2005;120(2):169-181.

15. Barski A, Cuddapah S, Cui K, et al. High-resolution profiling of histone methylations in the human genome. *Cell*. 2007;129(4):823-837.

16. Ketkar S, Verdoni AM, Smith AM, et al. Remethylation of Dnmt3a (-/-) hematopoietic cells is associated with partial correction of gene dysregulation and reduced myeloid skewing. *Proc Natl Acad Sci U S A*. 2020;117(6):3123-3134.

17. Petti AA, Williams SR, Miller CA, et al. A general approach for detecting expressed mutations in AML cells using single cell RNA-sequencing. *Nat Commun*. 2019;10(1):3660.

18. Robinson MD, McCarthy DJ, Smyth GK. edgeR: a Bioconductor package for differential expression analysis of digital gene expression data. *Bioinformatics*. 2010;26(1):139-140.

19. Jacquart A, Brayner R, El Hage Chahine JM, Ha-Duong NT. Cd(2+) and Pb(2+) complexation by glutathione and the phytochelatins. *Chem Biol Interact*. 2017;267:2-10.
