# Supplementary material for: Kdm6a deficiency restricted to mouse hematopoietic cells causes an age- and sex-dependent myelodysplastic syndrome-like phenotype
Source: PLoS One. 2021 Nov 15;16(11):e0255706. doi: 10.1371/journal.pone.0255706 (PMC8592440; doi:10.1371/journal.pone.0255706)
Supplement: S1 Raw images — (PDF) [file pone.0255706.s014.pdf]

A

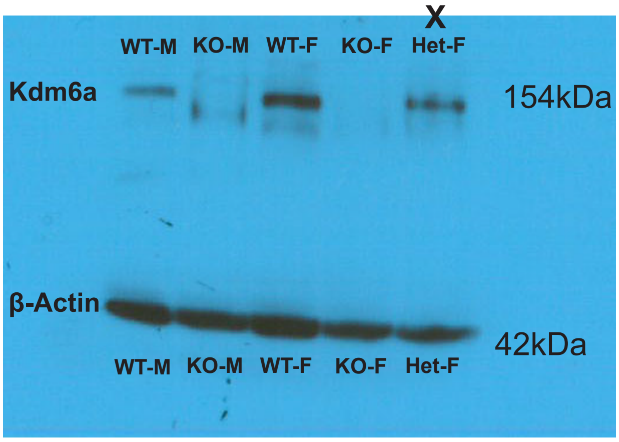

B

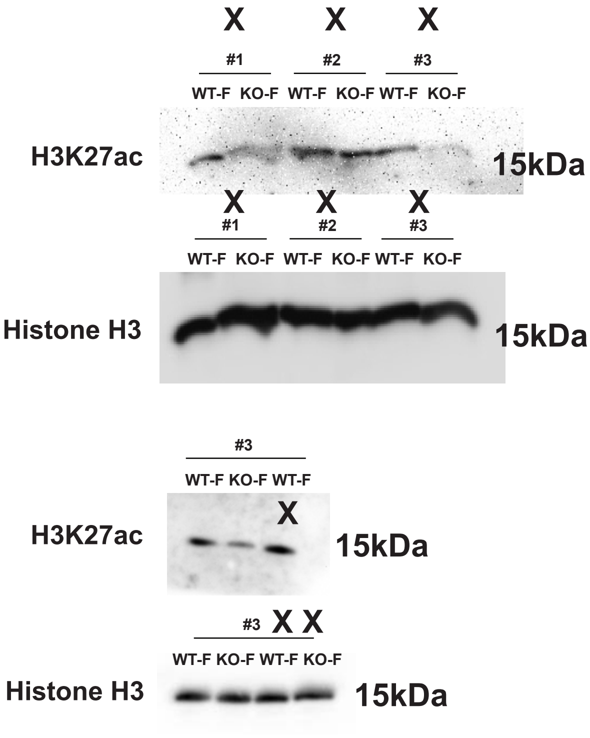

* #1, #2 and #3 represent 3 pairs of young female homozygous Kdm6a KO mice and littermate controls

Raw image file: A) Western blot analysis of protein lysate from whole bone marrow cells. This image was used to make Figure 1B. **B)** Western blot of core histone protein lysates used for Figure S7. The top panels shows three pairs of mice (WT-F and KO-F). The bottom panel demonstrates pair number three, which was used to prepare the manuscript.
