# Supplementary material for: Kdm6a deficiency restricted to mouse hematopoietic cells causes an age- and sex-dependent myelodysplastic syndrome-like phenotype
Source: PLoS One. 2021 Nov 15;16(11):e0255706. doi: 10.1371/journal.pone.0255706 (PMC8592440; doi:10.1371/journal.pone.0255706)
Supplement: Attachment — Submitted filename: Response to the Reviewers.docx [file pone.0255706.s016.docx]

**Point-by-point Response**

Dear Dr. Wutz,

We want to take the time to thank you for your investment in the review process. Based on your careful assessment we have made the following changes:

**1) Line 374 the figure S7A-C reference does not show an immunoblot of H3K27ac. Please, check the figure numbering and references in the text match up.**

We apologize for the oversight and thank the editor for bringing this inconsistency to our attention. After adding figure S5 to address reviewer comments our numbering did not adjust properly. In the current version all supplemental figure legends, text, and figure match up.

**B) Figure S6A shows survival curves for different genotypes. The curves of all but the control (black) are not obvious which might be caused by overlap. Please, check that this figure is a correct representation of the data.**

We thank the editor for raising this point. For figure S6A there was no differences between in the leukemia free percent survival curves for each genotype. When these data are plotted on Prism Graph Pad, the overlap between genotypes causes there to be only one line observed. We have gone back to the original files and can confirm that these data include all genotypes. To make these data more clear we have changed the title of the Y-axis to “leukemia free percent survival” and have included censored data of mice that died of other causes not related to a leukemia or known malignancy.
